# Glycine Decarboxylase Regulates Renal Carcinoma Progression via Interferon Stimulated Gene Factor 3-Mediated Pathway

**DOI:** 10.7150/ijbs.104458

**Published:** 2025-01-01

**Authors:** Thi Tuyet Mai Pham, Mikyung Kim, Thuy Quynh Nhu Nguyen, Jae-Hyung Park, Jee In Kim, Ji Hae Seo, Jin Young Kim, Eunyoung Ha

**Affiliations:** 1 Department of Biochemistry, School of Medicine, Keimyung University, Daegu 42601, Republic of Korea.; 2 Department of Physiology, School of Medicine, Keimyung University, Republic of Korea.; 3 Department of Molecular Medicine, School of Medicine, Keimyung University, Republic of Korea.; 4 Division of Haematology and Oncology, Department of Internal Medicine, School of Medicine, Keimyung University, Republic of Korea.

**Keywords:** GLDC, renal cell carcinoma, nucleotide synthesis, oxidative stress, interferon stimulated gene factor 3

## Abstract

Renal cell carcinoma (RCC) is considered as a “metabolic disease” due to various perturbations in metabolic pathways that could drive cancer development. Glycine decarboxylase (GLDC) is a mitochondrial enzyme that takes part in the oxidation of glycine to support nucleotide biosynthesis via transfer of one-carbon units. Herein, we aimed to investigate the potential role of GLDC in RCC development. We found that GLDC depletion diminished nucleotide synthesis and promoted reactive oxygen species (ROS) generation to repress RCC progression, which was reversed by repletion of deoxynucleosides. Additionally, *in vitro* and *in vivo* studies revealed that GLDC plays an important role in regulation of proliferation and tumor growth via interferon stimulated gene factor 3 (ISGF3)-mediated pathway. Expressions of interferon regulatory factor 9 (IRF9) and signal transducer and activator of transcription 2 (STAT2) were elevated in GLDC knock-downed cells and decreased in GLDC over-expressed cells. Double knock-down of STAT2 and IRF9 in GLDC-deficient cells rescued GLDC depletion-induced decrease in cell proliferation. Furthermore, GLDC depletion increased cisplatin-and doxorubicin-induced DNA damage through ISGF3 pathway, leading to cell cycle dysregulation and increased mitotic catastrophe. These findings reveal that GLDC regulates RCC progression via ISFG3-mediated pathway and offers a promising strategy for RCC treatment.

## Introduction

Renal cell carcinoma (RCC) is one of the most prevalent cancers, marked by rising incidence rates and high mortality rates. The mortality of RCC varies mostly depending on cancer stages. Early stage of RCC presents a relatively higher survival rate compared to advanced or metastatic RCC. Although there are improvements in diagnosis and treatment, the overall prognosis of RCC remains unfavorable 1, 2. Recently, treatment of RCC has shifted from non-specific immune approach to inhibition of target molecules 1. Therefore, finding a potential therapeutic target for treatment of RCC needs to be investigated.

Nucleotides supply substrates essential for numerous cellular processes including cell proliferation and replication. Nucleotide pools in proliferating cells increase 5 -10 times more than those in resting cells 3. A previous study has shown that mammalian target of rapamycin (mTOR) signaling, a key driver of proliferation, can stimulate nucleotide synthesis via increasing the activity of key enzymes in *de novo* pyrimidine synthesis 4. As such, the modulation of enzymes involved in the nucleotide metabolism could alter deoxynucleotide triphosphate (dNTP) levels, thereby either suppressing or stimulating cell proliferation and replication. Various human cancer cells exhibit elevated dNTP levels compared to non-malignant cells 5. Particularly, canonical driver genes of human cancer, such as MYC, can upregulate nucleotide synthesis by directly or indirectly inducing genes in nucleotide synthesis 6. Depletion of dNTPs not only impairs cell proliferation but also affects cellular response to chemotherapy and homeostasis 7-10. Hence, targeting genes in nucleotide synthesis can be an effective strategy for development of cancer therapeutics.

Glycine decarboxylase (GLDC) is a part of mitochondrial complex enzyme, glycine cleavage system (GCS). GCS cleaves glycine to produce an intermediate that later generates 5,10-methylene tetrahydrofolate (5,10-meTHF), an intermediate of one-carbon metabolism. One-carbon metabolism is a well-established metabolic pathway that plays a part in *de novo* pyrimidine biosynthesis 11. The role of GLDC in cancer development and progression has only recently been elucidated and appears complex and controversial. A previous study showed that GLDC acts as an oncogene in non-small cell lung cancer 12 and another study demonstrated that GLDC is a tumor suppressor in hepatocellular carcinoma 13. Also recent studies revealed that GLDC promotes metastasis of colorectal cancer 14 and plays a crucial role in the progression of prostate cancer 15. Hence, the role of GLDC in cancer development and progression seemingly differs amongst different cancer types.

Interferon stimulated gene factor 3 (ISGF3) is a complex of signal transducer and activator of transcription 1 and 2 (STAT1 and STAT2) and interferon regulatory factor 9 (IRF9) 16. STAT1, STAT2, and IRF9 are assembled into ISGF3 in respond to various stimuli including interferons (IFNs) and DNA damages 16. ISGF3 complex drives the transcription of a subset of IFN-stimulated genes (ISGs), which regulate multiple pathways including cell proliferation, cell death, antiviral response, and senescence. STAT1, STAT2, and IRF9 are themselves ISGs, and can activate a subset of ISGs 16, 17. Our understanding of the function of ISGF3 in cancer development and progression remains incomplete. A recent study suggested that ISGF level is commonly regulated by polybromo 1 (PBRM1), SET domain containing 2 (SETD2), lysine demethylase 5C (KDM5C), and BRCA1 associated protein 1 (BAP1), tumor suppressor genes that are secondarily mutated in RCC after VHL (von Hippel-Lindau) inactivation 18. Another study showed that ISGF3 can be induced by mitochondrial stress, especially mitochondrial DNA (mtDNA) 19.

In this study, we tested our hypothesis that GLDC affects *de novo* nucleotide production that may lead to inhibition/or stimulation of RCC progression possibly via ISGF3-mediated pathway.

## Materials and methods

### Cell culture

The human cell lines, ACHN, Caki-2, HK2, and A498 were obtained from the American Type Culture Collection (ATCC). Cells were cultured in appropriate medium supplemented with 10% fetal bovine serum (FBS) and 1% penicillin-streptomycin in incubator (37 ^0^C and 5% CO_2_). ACHN and Caki-2 cells were maintained in DMEM high glucose medium (WELLGENE, Gyeongsangbuk-do, Republic of Korea). HK2 cells were maintained in RPMI medium (WELLGENE, Gyeongsangbuk-do, Republic of Korea). A498 cells were maintained in DMEM F12 medium (Gibco, MT, USA).

### Reagents

Lentivirus for overexpression of GLDC (OE GLDC) and corresponding control vector (EV) were purchased from Addgene, MA, USA. Lentivirus for short hairpin RNA (shRNA) of GLDC (shGLDC) and corresponding control vector (CTL) were purchased from Sigma-Aldrich, MA, USA. Short interfering RNAs (siRNAs) for STAT2 and IRF9 came from Bioneer, Republic of Korea. Deoxynucleosides (dNs) including deoxyadenosine (dA), deoxythymidine (dT), deoxycytidine (dC), and deoxyguanosine (dG) were purchased from Sigma-Aldrich, MA, USA. To prevent the degradation of deoxyadenosine triphosphate (dATP), erythro-9-(2-hydroxy-3-nonly) adenine (EHNA) (Tocris, Bristol, UK) was added to the medium 20. Brequinar (Bre) sodium was purchased from Tocris, Bristol, UK. All of the followings were described in the supplement section: primers for polymerase chain reaction (PCR) (Table S1), DNA oligonucleotides (Table S2), and short interfering RNAs (siRNA) (Table S3).

### RCC clinical samples

Pair RCC tissues and non-tumor tissues (25 pairs) were collected from Dongsan Hospital, Daegu, Korea. All sample donors provided informed consent as required by the Institutional Review Board at Keimyung University (School of Medicine). The study was conducted with ethical approval from Keimyung University Hospital ethics committee (#2020-10-068).

### Establishment of stable cell lines and transfection of cells

To produce lentivirus, plasmids were co-transfected with packaging plasmid into HEK293T cells for 48 h. The supernatant containing lentivirus particles were collected and infected to target cells with polybrene. To select the stably transfected cells, puromycin was used at concentrations of 1-10 μg/mL in culture media. Puromycin containing fresh media was added every 3-4 days until colonies became visible. Cells were collected and protein lysates and total RNA were extracted to verify the efficiency of transfection. siRNAs for STAT2 and IRF9 were transfected to cells using Oligofectamin (12252011, Invitrogen, USA) or Lipofectamin 2000 (11668-019, Invitrogen, USA). Protein lysates and total RNA were collected 24 or 48 h after to verify the efficiency of transfection by quantitative polymerase chain reaction (qPCR) and western blot.

### Cell proliferation and viability assay

Cells were plated into 96 well-plates. The proliferation rate was assessed at 0, 24, 48, and 96 h using Cell Counting Kit-8 (CCK8) (Dojindo Molecular Technologies, Kumamoto, Japan) assay. Absorbance at 450nm wavelength was measured. For viability assay, CCK8 was added to assess cell viability 12, 24, and 48 h after treatment.

### Colony formation

Cells were plated into 6-well/or 12-well plates at triplicate, collected 1-2 weeks after seeding, and fixed with 4% paraformaldehyde (PFA) (J19943-K2, Thermo-Scientific, MA, USA) for 15 min. Colonies were stained with 0.05% crystal violet for 15 min and washed with phosphate-buffered saline (PBS). To quantify staining intensity, methanol was added for 5 min and wavelength at 570 nm were measured.

### Migration assay

Migration and invasion assay were performed using transwell chambers (Corning, MA, USA. Cells in serum-free medium were added to upper chamber. DMEM with 10% FBS was added to the lower chamber. Migrated or invaded cells were fixed 24 h after with 4% PFA and stained with 0.05% crystal violet. The number of cells migrated was counted under the microscope.

### Wound healing assay

Cells were grown in 6/or 12 well-plates. When the confluence reached 80-90%, the wound was created by scratching with 10 µl pipette tip. Then cells were washed and added fresh medium for the next 24 h. Images were captured at 0 h and 24 h, respectively. Wound areas were calculated using ImageJ.

### Western blot

For western blot assay, cells and tissues were lysed by radioimmunoprecipitation assay buffer (RIPA buffer) (EBA-1149, Elpis Biotech, Daejeon, Republic of Korea) containing protease inhibitor cocktail and phenylmethylsulfonyl fluoride (PMSF). Same amounts of proteins were loaded into sodium dodecyl sulfate polyacrylamide gel electrophoresis (SDS-PAGE). Proteins were separated and transferred to polyvinylidene fluoride (PVDF) membrane. Then, the membrane was incubated with indicated antibodies and developed with chemiluminescence. Antibodies: β-actin (4967S, Cell Signaling, Massachusetts, USA), GAPDH (GTX627408, GeneTex, CA,USA), GLDC (PA5-22102, Thermo Fisher, MA, USA), IRF9 (76684, Cell Signaling, MA, USA), STAT2 (44-362G, Thermo Fisher, MA, USA), STAT1 (AHO0832, Thermo Fisher, MA, USA), SETD2 (PA5-34934, Thermo Fisher, MA, USA), H3K36me3 (ab9050, Abcam, Cambridge, UK), H3 (4620, Cell Signaling, MA, USA), and p-H2AX (9718, Cell Signaling, MA, USA).

### RNA isolation and qPCR

TRIzol reagent (Ambion, CA, USA) was used to extract total RNA of cells following the instructions of the manufacturer. The NanoDrop 2000 spectrophotometer (NanoDrop Technologies, Wilmington, USA) was used to measure the purify and the concentration of RNA. qPCR was performed with SYBR Green mix (TOYOBO, MI, USA) using LightCycler 480 II, and normalized to β-actin.

### Immunofluorescence (IF) staining

Cells were fixed with 4% PFA, permeabilized with 0.1% TritonX-100 for 10 min. After blocking with 1% bovine serum albumin (BSA), cells were stained with primary antibody against p-H2AX (9718, Cell Signaling, MA, USA), lamin B1(ab16048, Abcam, Cambridge, UK), DNA (CBL186, Sigma-Aldrich, MA, USA), and HSP60 (12165, Cell Signaling, MA, USA) for overnight at 4 °C. Secondary antibody was applied for 1 h at room temperature. Nuclei were stained with 4',6-diamidino-2-phenylindole (DAPI). Cells were observed under Leica Stellaris 5 confocal microscope. For quantification of nucleoid area, approximately 5-10 confocal images comprising between 50-100 nucleoids were randomly captured. ImageJ was used to measure the nucleoid area. Nucleoids were divided into three sizes: <200 nm^2^, 200-450 nm^2^, and >450 nm^2^.

### Measurements of reactive oxygen species (ROS)

The level of intracellular ROS was measured with mitoSOX (Thermo Fisher, MA, USA) staining according to the manufacturer's instruction. Pictures were taken randomly under Leica Stellaris 5 confocal microscope and analyzed for fluorescence intensity by ImageJ software.

### Animal experiment

Seven-week-old male BALB/c nude mice were purchased from the JA Bio (Korea). Subcutaneous injections were performed with ACHN and A498 cells in matrigel. Caliper was used to measure tumor growth twice a week. Tumor volumes were evaluated with the formula of V = 1/2 L × W^2^ (V: tumor volume, L: tumor length, W: tumor width). Tumor weights were measured on the day mice were sacrificed. All animal experiments were approved by the Institutional Animal Care and Use Committee (IACUC) of Keimyung University, School of Medicine, Daegu, Korea (KM-2020-16R1).

### Immunohistochemistry (IHC)

Four μm thick paraffin slides were deparaffinized and rehydrated. Antigen retrieval was performed with citrate buffer (pH=6.0) for 20 min. Slides were blocked with goat serum for 30 min followed by staining with primary antibody for overnight at 4 °C. Then, secondary antibody was applied for 30 min at room temperature. The positive signal was developed using diaminobenzidine chromogen (DAB) (SK-4105, Vector Laboratories, USA) and counterstained with hematoxylin. Slides were observed with Leica microscope (magnification 400X).

### Deoxynucleotide triphosphates (dNTPs) extraction and measurement

Cells were seeded in 75-disk flask and were allowed to enter log-phase growth for 48 h. Then, cells were counted, suspended in 500 μL of ice-cold 60% methanol and sonicated for 30 s in a Brandson Sofifier SFX 550 (Branson, CT, USA). The lysates were incubated at 95 °C for 3 min and centrifuged 18,500 x g for 6 min at 4 °C to remove cell pellet. The supernatants were poured onto Amicon Ultra-0.5-ml centrifugal filters (Merck Millipore, MA, USA) and washed twice with diethyl ether. The extracts were stored at -80 °C until use. Detailed method for measurement of dNTPs was the same as described previously 21.

### Statistical analysis

All the experiments were performed three times independently. All analyses were performed with GraphPad Prism 8.2.0. The results were represented as mean ± SD. Statistical analysis was conducted using Student's *t*-test or one-way ANOVA for more than two groups. p-value <0.05 was considered statistically significant.

## Results

### Suppression of GLDC induces dNTPs depletion, resulting in ROS accumulation and leading to mitochondrial stress in RCC cells

**Figure 1:**
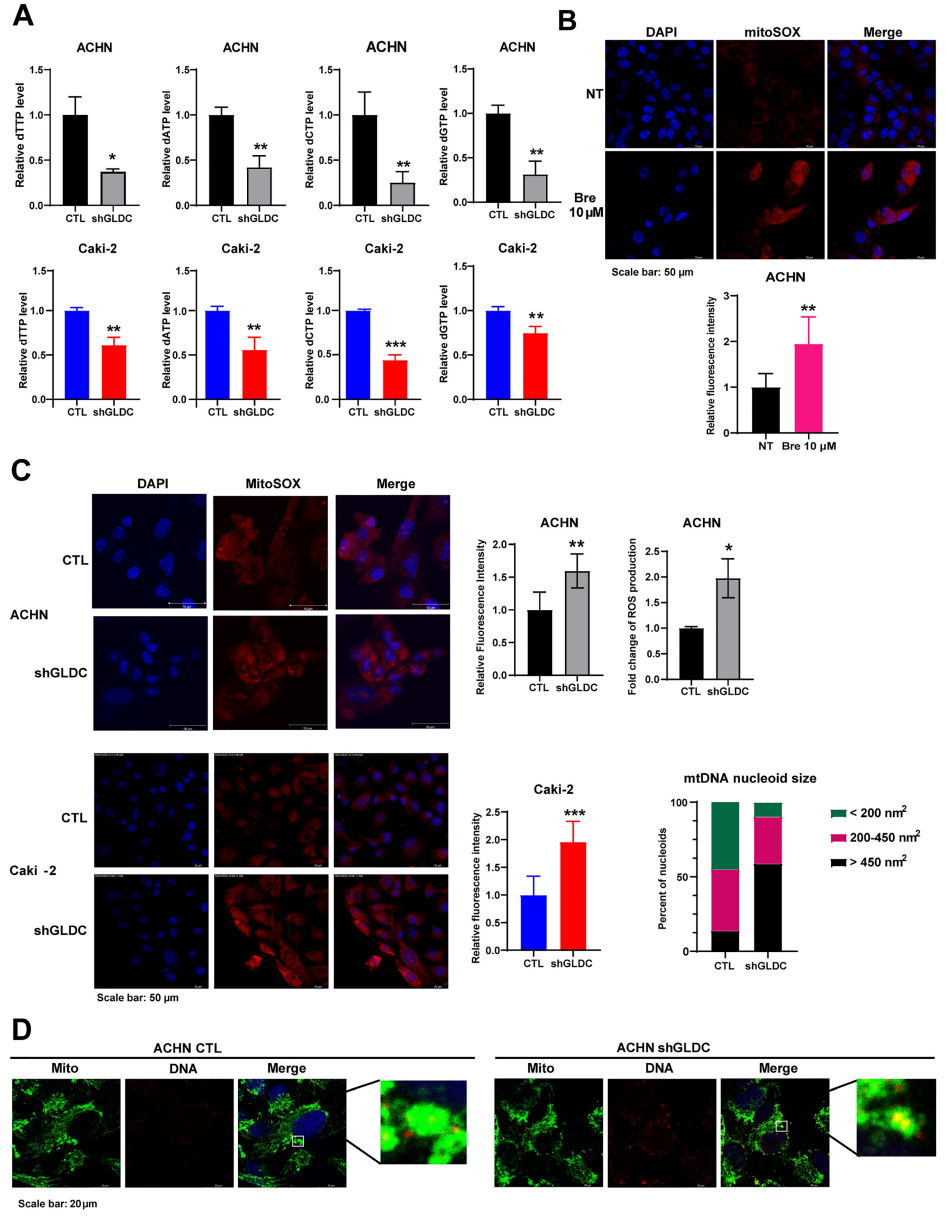
** Suppression of GLDC induces dNTPs depletion, resulting in ROS accumulation and leading to mitochondrial stress in RCC cells. (A)** Quantification of dTTP, dATP, dCTP, and dGTP levels in ACHN and Caki-2 cells. CTL, control; shGLDC, knockdown of GLDC. Data are shown as the means ± SD (*n*=3). **(B)** Representative micrographs of immunofluorescence staining for mitoSOX with 4',6-diamidino-2-phenylindole (DAPI) counterstaining. ACHN cells were treated with brequinar (Bre) 10 µM for 48 h. ImageJ is used for quantification of the fluorescence intensity. NT, non-treated cells. Data are shown as the means ± SD (*n*=3). **(C)** Representative micrographs of immunofluorescence staining for mitoSOX with DAPI counterstaining in control (CTL) and GLDC knock-downed (shGLDC) cells. The quantifications of fluorescence intensity are on the right side. Data are shown as the means ± SD (*n*=3). MitoSOX staining intensity in ACHN CTL and shGLDC cells was confirmed by FACS analysis. Data are shown as the means ± SD (*n*=3). **(D)** Representative micrographs of immunofluorescence staining for mitochondria (anti-HSP60) and mitochondrial (mt) DNA nucleoids (anti-DNA). ImageJ was used to calculate the size of mtDNA nucleoids. Mito, mitochondria. p values were calculated using two-tailed unpaired Student *t*-test. *p < 0.05, **p < 0.01, and ***p < 0.001.

GLDC is the enzyme that catalyzes the first and rate limiting step in glycine metabolism, the product of which is used for nucleotide synthesis via the tetrahydrofolate (THF) cycle of one carbon metabolism 22, 23. Therefore, we hypothesized that knockdown of GLDC would attenuate *de novo* nucleotide synthesis eventually decreasing intracellular dNTP pools. We first determined the levels of dNTP in control and GLDC knock-downed cells. Fig. 1A shows that knockdown of GLDC decreased levels of all four types of dNTP in both ACHN and Caki-2 cells. Previous studies have shown that inhibition of nucleotide synthesis via silencing of dihydroorotate dehydrogenase (DHODH) causes ROS accumulation 24, 25. Accordingly, we determined to assess ROS production in RCC cells and observed that Bre, a DHODH specific inhibitor, increased ROS production in ACHN cells (Fig. 1B). Consistent with this result, we found that knockdown of GLDC also increased ROS generation (Fig. 1C). These results show that GLDC knockdown diminishes *de novo* nucleotide synthesis resulting in increased ROS levels in RCC cells.

Augmented ROS level promotes mitochondrial dysfunction and induces mitochondria DNA (mtDNA) stress 26, 27. mtDNA stress is accompanied by enlarged nucleoid - DNA in mitochondria- and the escape of mtDNA to cytosol, where it promotes cGAS-STING signaling to elevate expressions of ISGs 19, 28. To explore the possible effect of GLDC on mtDNA, we performed double-IF staining of heat shock protein 60 (HSP60), a mitochondrial marker, and DNA in ACHN control and GLDC knock-downed cells. The results suggested that depletion of GLDC in ACHN cells induced enlarged nucleoids (Fig. 1D). These results suggest that GLDC depletion-induced decrease in dNTP synthesis could lead to elevated ROS production and elevated ROS may induce mtDNA stress.

### Knockdown of GLDC inhibits cell proliferation, colony formation and sphere formation

To evaluate the function of GLDC, we successfully constructed ACHN and Caki-2 in which GLDC is stably knock-downed by transfecting short hairpin RNA (shRNA) and HK2 and A498 in which GLDC is stably over-expressed by transfecting lentivirus.

**Figure 2:**
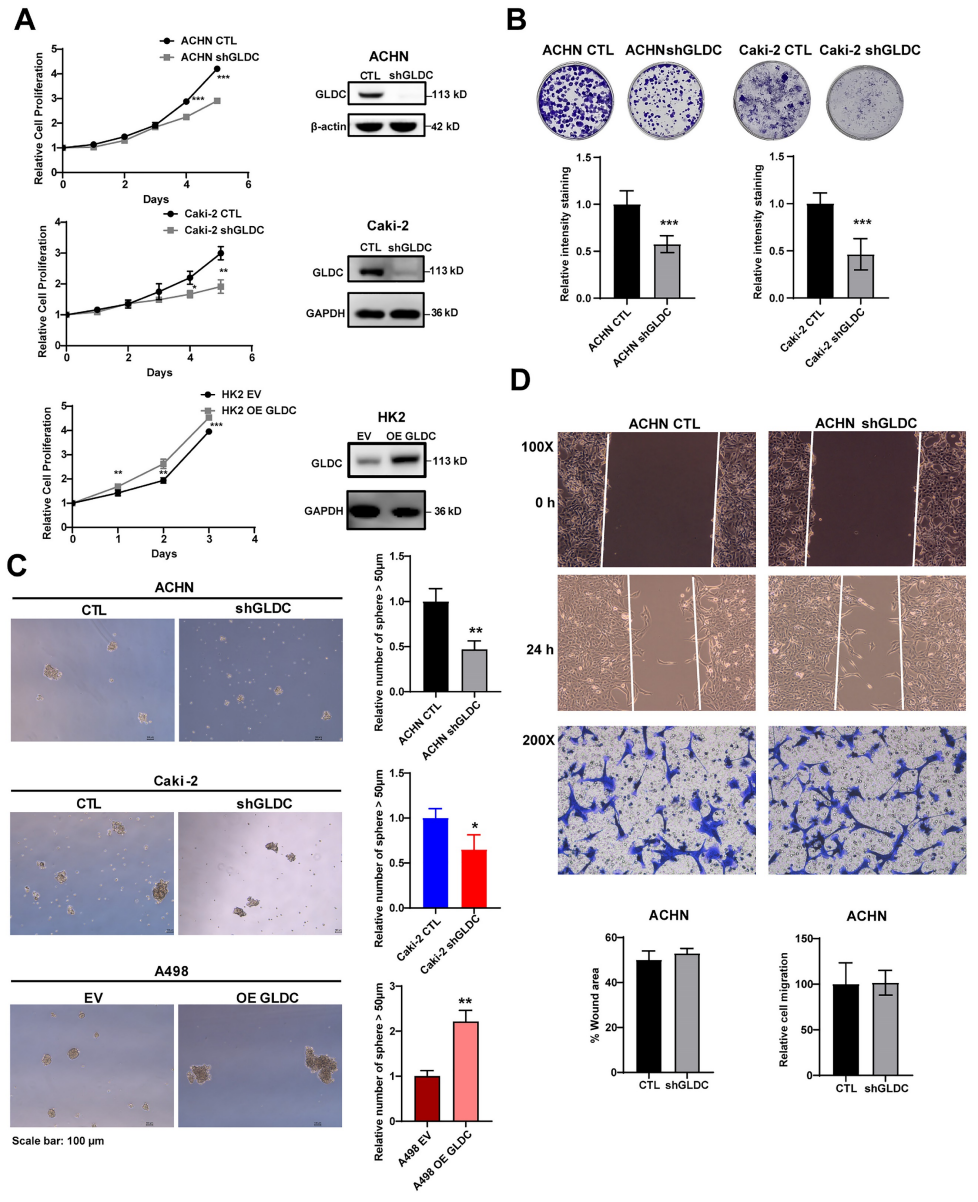
** Knockdown of GLDC inhibits cell proliferation, colony formation and sphere formation. (A)** Cell proliferation of indicated cell lines was assessed using CCK-8 assay. Successful knockdown (shGLDC) or overexpression of GLDC (OE GLDC) were confirmed by western blot on the right side. Data are shown as the means ± SD (*n*=3). **(B)** Colony formation of indicated cell lines. The intensity of staining was quantified by dissolving cells with methanol for 5 min and measuring absorbance at 570 nm wavelength. Data are shown as the means ± SD (*n*=3). **(C)** Primary sphere formation of indicated cell lines. The number of spheres were calculated under microscope, and the sizes of spheres were determined using ImageJ (*n*=3). **(D)** Upper panel; wound healing assay of ACHN CTL and shGLDC cells. Wound areas were captured at 0 h and 24 h post-scratching and quantified by ImageJ (*n*=3). Lower panel; cell migration assay of indicated cells. Migrated cells were observed under a microscope at 200X magnification and calculated (*n*=3). p values were calculated using two-tailed unpaired Student t-test. *p < 0.05, **p < 0.01, and ***p < 0.001. CTL, control; EV, corresponding control for OE GLDC.

The results showed that proliferation rate was markedly lower in GLDC knock-downed cells than that in control cells. Conversely, overexpression of GLDC increased cellular proliferation in HK2 proximal tubular cells (Fig. 2A). The colony formation assay showed corroborating results with decreased colony formations in ACHN and Caki-2 GLDC knock-downed cells (Fig. 2B). We also found that the number of primary spheres larger than 50 µm was lower in GLDC knock-downed cells than that in control cells while the number of primary spheres larger than 50 µm was higher in GLDC over-expressed cells than that in the control (Fig. 2C). Moreover, depletion of GLDC in ACHN cells indeed attenuated the number of secondary tumor spheres and overexpression GLDC in A498 cells stimulated the number of secondary tumor spheres (Fig. S1A). However, we found that depletion of GLDC in ACHN cells didn't affect the cell migration and invasion performed by wound healing and invasion assay (Fig. 2D). In summary, these results indicate that GLDC regulates cellular proliferation and colony formation.

### GLDC regulates RCC cell progression via ISGF3 pathway

**Figure 3:**
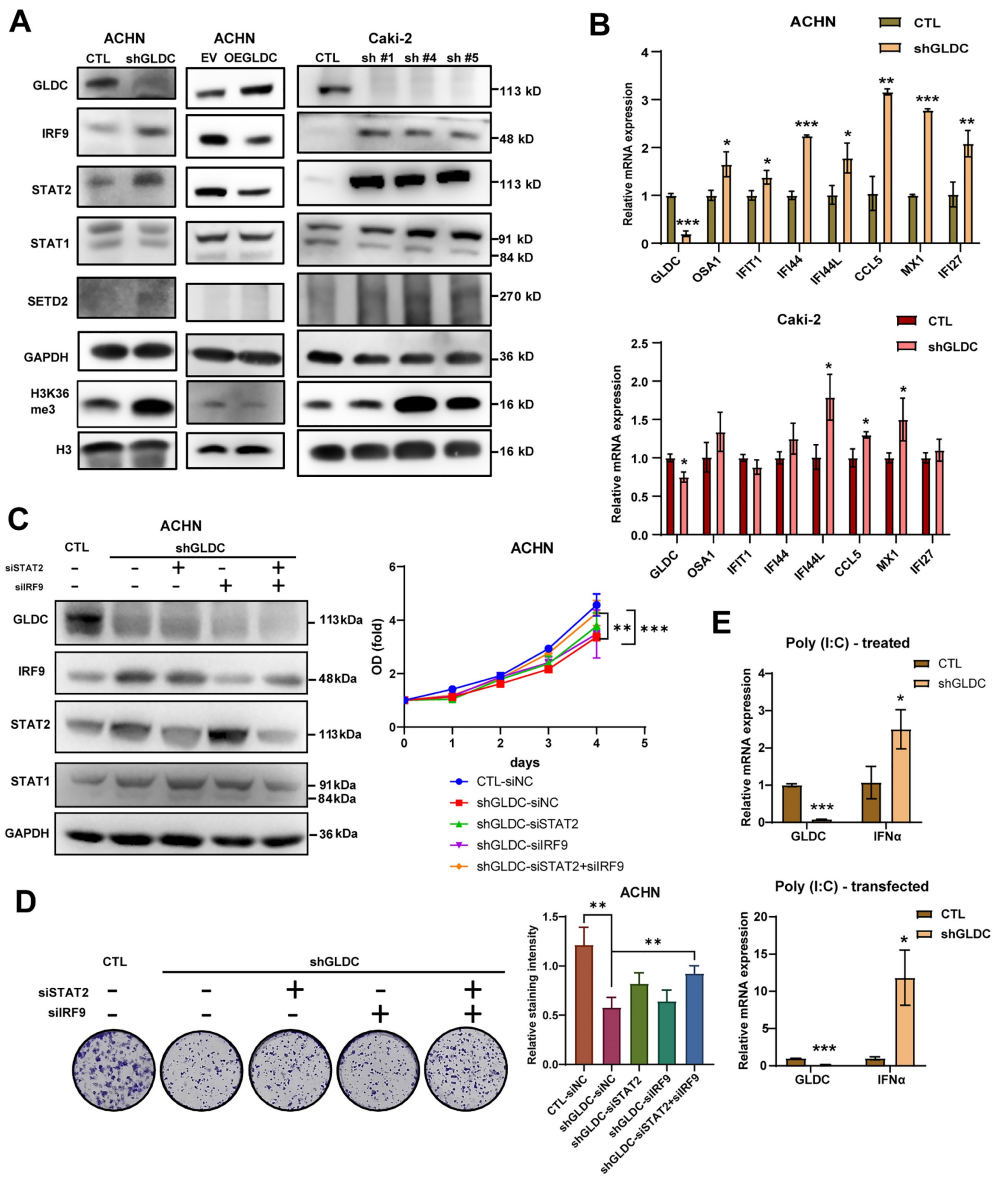
** GLDC regulates RCC cell progression via ISGF3 pathway. (A)** Western blot of GLDC, IRF9, STAT2, STAT1, SETD2, GAPDH (loading control), H3K36me3, and H3 (loading control for H3K36me3) in indicated cells. To confirm in Caki-2 cells, we suppressed the expression of GLDC in Caki-2 cells with three shRNA constructs (shGLDC #1, shGLDC #4 and shGLDC #5), and observed the expression of indicated proteins. **(B)** Quantitative PCR (qPCR) analysis of the ISGs: OSA1, IFIT1, IFI44, IFI44L, CCL5, MX1, and IFI27 of indicated cells. Data are shown as the means ± SD (*n*=3).** (C)** Western blot analysis of indicated proteins in ACHN CTL and shGLDC cells transfected with siRNA of negative control (siNC), STAT2 and/ or IRF9 in ACHN knock-downed GLDC cells compared to control (siNC)-siRNA cells. The cellular growth of indicated cells was on the right side (*n*=3).** (D)** Colony formation of indicated cells. Quantification of staining intensity was performed at a wavelength of 570 nm (*n*=3). **(E)** qPCR analysis of IFN-α in ACHN CTL and shGLDC treated with Poly (I:C) (10 µg) or transfected with Poly (I:C) (1 µg) for 24 h. Data are shown as the means ± SD (*n*=3). p values were calculated using two-tailed unpaired Student t-test. *p < 0.05, **p < 0.01, and ***p < 0.001. CTL, control; shGLDC, knockdown of GLDC; EV, corresponding control for OE GLDC; OE GLDC, overexpression of GLDC.

To elucidate the underlying mechanism of the effect of GLDC on RCC progression, we chose ACHN and Caki-2 with stable knock-downed GLDC and conducted further experiments. Based on previous studies showing that mtDNA stress activates innate immune systems via unphosphorylated-ISGF3 (U-ISGF3) and that ISGF3 acts as a tumor suppressor in RCC 16, 18, 29, we hypothesized that GLDC could regulate RCC progression via ISGF3. ISGF3 is a transcription factor composed of STAT1, STAT2 and IRF9 and can be activated by increased expression or phosphorylation of its subunits, STAT1 and STAT2 30. We first determined the expression levels of ISGF3 subunits, STAT1, STAT2, and IRF9. Here, we found that the knockdown of GLDC in ACHN cells increased levels of STAT2 and IRF9 but not STAT1. Conversely, expression levels of STAT2 and IRF9 decreased in GLDC overexpressed ACHN cells (Fig. 3A). We also suppressed the expression of GLDC in Caki-2 cells with three shRNA constructs (shGLDC #1, shGLDC #4 and shGLDC #5), and found that increased expression levels of IRF9, STAT1 and STAT2 in Caki-2 GLDC knock-downed cells (Fig. 3A). Corroborating with these results, we observed increased levels of SETD2, a transcription factor that regulates ISGF3, and downstream target of SETD2, histone 3 lysine 36 trimethylation (H3K36me3) in GLDC deficient ACHN and Caki-2 cells. Moreover, we found increased mRNA expressions of ISGF3 target genes in GLDC knock-downed ACHN and Caki-2 cells (Fig. 3B).

Next, we considered whether suppression of GLDC could lead to phosphorylation of STAT2. And we found that STAT2 was not phosphorylated by suppression of GLDC (Fig. S1B). This result suggests that increased level unphosphorylated-ISGF3, not phosphorylated ISGF3, is responsible for the activation of ISGF3 pathway. To further clarify the function of ISGF3-mediated effects of GLDC on cell proliferation and colony formation, we transfected siRNAs of STAT2 and/or IRF9 in GLDC knock-downed cells and observed that silencing of STAT2 or IRF9 alone did not reverse cell proliferation and colony formation but double silencing of STAT2 and IRF9 reversed both cell proliferation and colony formation (Fig. 3C-D). These results show that GLDC knockdown regulates RCC progression possibly via ISGF3-mediated pathway, especially increasing expressions of ISGF3 subunits, STAT2 and IRF9.

Given the previous result that GLDC plays a crucial role in viral infection 31, we reasoned that GLDC-ISGF3-ISG axis could be induced by viral infection. To test this reasoning, we stimulated cells with polyinosinic-polycytidylic acid [poly(I:C)], a dsRNA analog that mimics viral infection and found poly(I:C) markedly increased interferon α (IFNα) in GLDC deficient cells compared with that in control cells (Fig. 3E).

### GLDC knockdown-induced depletion of dNTP inhibits cellular proliferation

**Figure 4:**
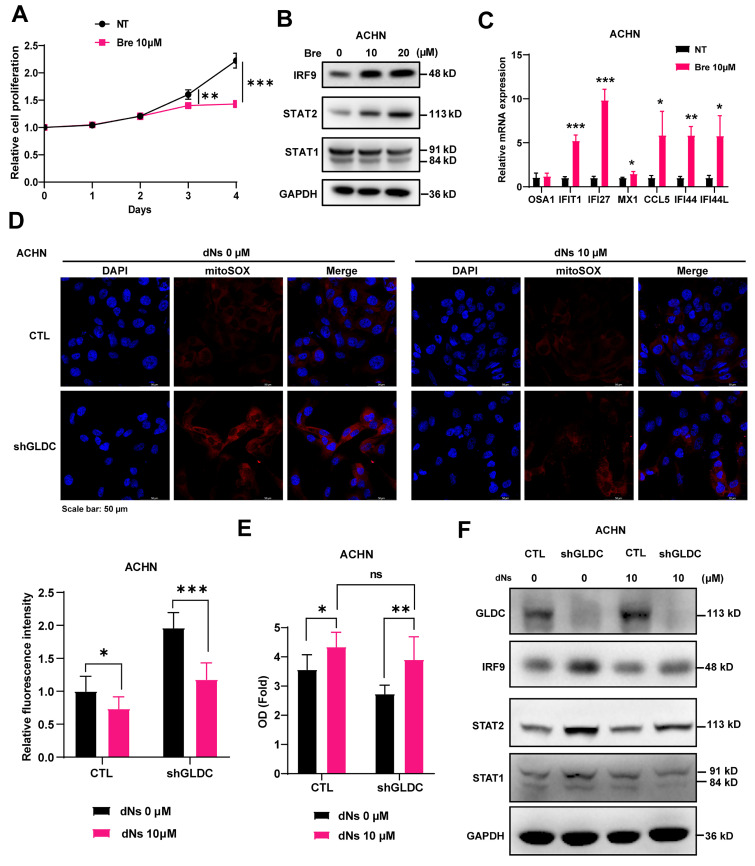
** GLDC regulates RCC cell proliferation by activating ISGF3 through the inhibition of dNTP synthesis. (A)** Growth curve of ACHN cells treated with brequinar (Bre) 10 µM or without (NT) was assessed by CCK-8 assays. Data are shown as the means ± SD (*n*=3).** (B)** Western blot of indicated proteins in ACHN cells treated with Bre 10 µM or 20 µM for 48 h. **(C)** qPCR analysis of ISGs in ACHN treated with Bre 10 µM compared to non-treated control cells (NT). Data are shown as the means ± SD (*n*=3). **(D)** Representative micrographs of immunofluorescence staining for mitoSOX in indicated cells with or without the addition of each dNs 10 µM and EHNA 5 µM for 48 h. The fluorescence intensity was calculated by ImageJ (*n*=3). **(E)** Cellular growth of ACHN control (CTL) and GLDC knock-downed cells (shGLDC) after the addition of each dNs 10 µM and EHNA 5 µM to inhibit of degradation of dATP in cell culture. Data are shown as the means ± SD (*n*=3). **(F)** Western blot of indicated proteins in ACHN CTL and shGLDC cells 48 h after the addition of each dNs 10 µM and EHNA 5 µM. p values were calculated using two-tailed unpaired Student *t*-test. *p < 0.05, **p < 0.01, and ***p < 0.001.

To further verify the mechanistic details of GLDC, depletion of GLDC leading to the decreased synthesis of dNTPs, increased mitochondrial stress, and activation of ISGF3 in RCC, we treated cells with Bre and found that Bre treatment decreased proliferation of ACHN RCC cells (Fig. 4A). Additionally, Bre treatment increased protein expression levels of STAT2 and IRF9, but not STAT1, and mRNA expression levels of ISGF3 downstream target genes (Fig. 4B-C). Furthermore, addition of dNs reversed GLDC depletion-induced generation of ROS in ACHN cells (Fig. 4D). Lastly, we treated RCC cells with dNs to determine whether dNs reverse GLDC depletion-induced decrease in cellular proliferation and indeed we observed increased cellular proliferation in GLDC-depleted ACHN RCC cells treated with dNs (Fig. 4E). Furthermore, after adding dNs, the number of GLDC knock-downed cells did not significantly differ from control cells. Consistently, addition of dNs decreased protein expressions of STAT2 and IRF9 in GLDC knock-downed cells (Fig. 4F). In summary, these results show that depletion of nucleotide synthesis induced by knockdown of GLDC mediates decreased cellular proliferation via ISGF3 activation.

### Knockdown of GLDC aggravates doxorubicin (Dox) and cisplatin (CP)-induced DNA damage

**Figure 5:**
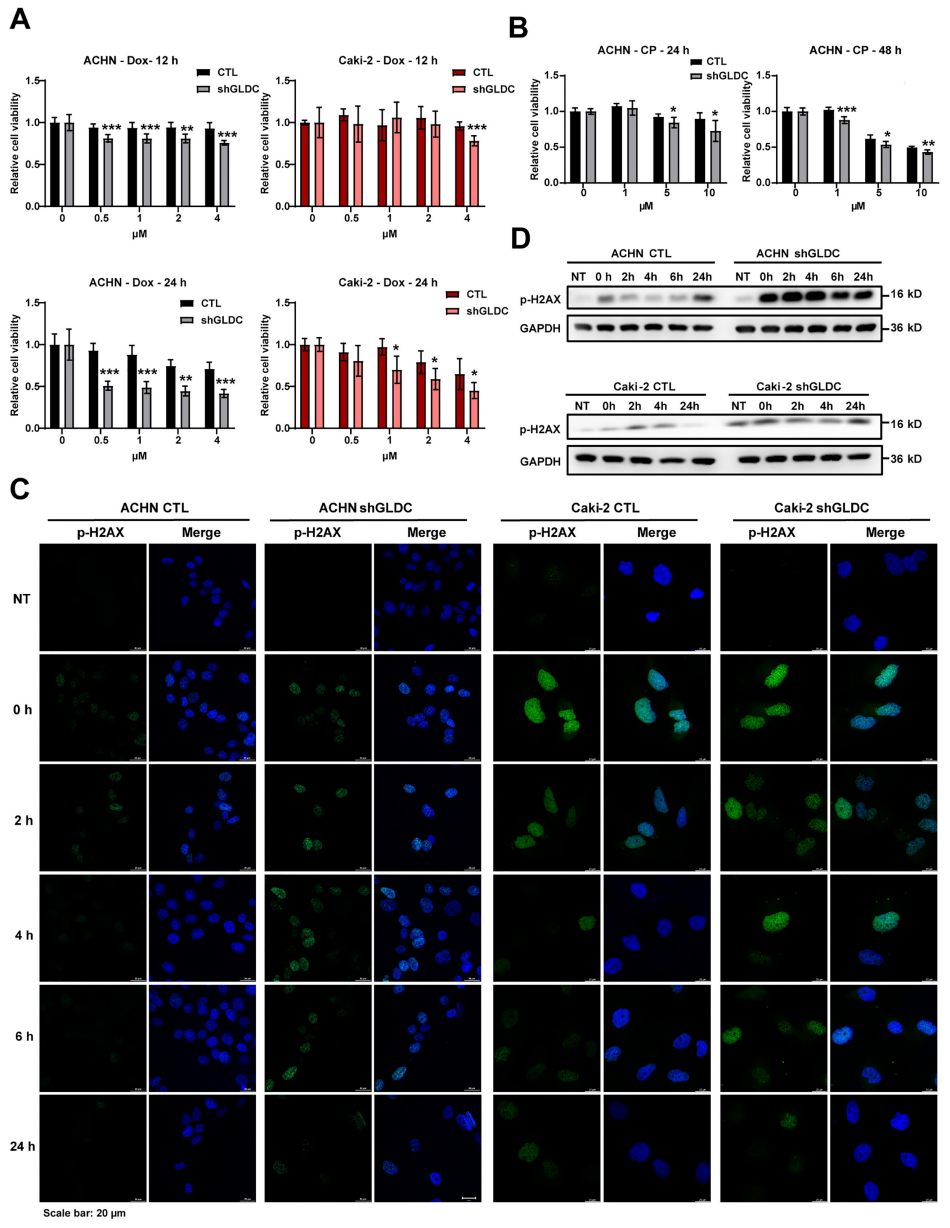
** Knockdown of GLDC aggravates doxorubicin (Dox) and cisplatin (CP)-induced DNA damage. (A)** Cell viability of indicated cell lines after treatment with doxorubicin (Dox) for 12 h or 24 h (*n*=3). **(B)** Cell viability of indicated cell lines after treatment with cisplatin (CP) for 24 h or 48 h (*n*=3). **(C)** Analysis of DNA damage and repair rate was conducted through the immunofluorescence staining of p-H2AX during recovery period following 12 h of treatment with Dox 1 µM - induced damage. NT, non-treated cells. **(D)** Western blot of p-H2AX in indicated cell lines during recovery period following 12 h of treatment with Dox 1 µM. p values were calculated using two-tailed unpaired Student *t*-test. *p < 0.05, **p < 0.01, and ***p < 0.001.

Given that inhibition of dNTP synthesis by anticancer drugs enhances double-strand breaks (DBSs) 10 and downregulation of GLDC inhibited dNTP synthesis and increased the expression of SETD2 that facilitates and repairs DSBs in RCC cells 32, we reasoned that GLDC might play a role in the cellular response to DNA damage. To test this hypothesis, we used anticancer drugs Dox and CP to induce DNA damage in ACHN and Caki-2 cells and determined whether GLDC knockdown affects cell viabilities in Dox/or CP-treated RCC cells. We indeed found that depletion of GLDC rendered cells more susceptible to Dox/or CP-induced cell death (Fig. 5A-B). Corroborating with this result, we also observed that expression of p-H2AX, a well-established DNA damage marker 33, increased earlier and lasted longer in GLDC knock-downed ACHN and Caki-2 cells than in control cells (Fig. 5C-D). These results suggest that knockdown of GLDC enhances Dox and CP-induced DNA damage and prolongs DNA repair process.

### Downregulation of ISGF3 subunits reverses Dox/and CP-induced DNA damage response in GLDC knock-downed cells

**Figure 6:**
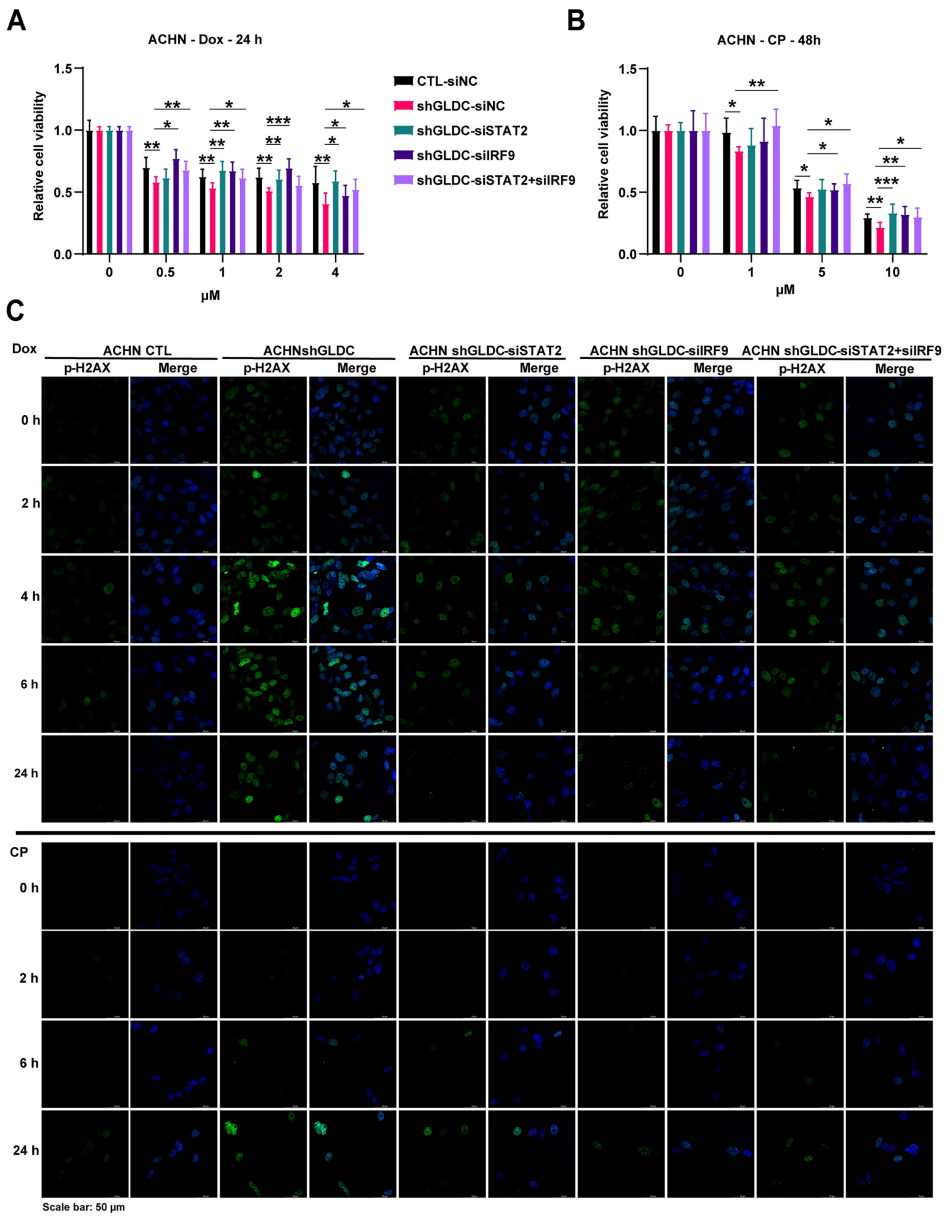
** Downregulation of ISGF3 subunits reverses Dox/and CP-induced DNA damage response in GLDC knock-downed cells. (A)** Cell viability of indicated cells with or without doxorubicin (Dox) treatment for 24 h. Data are shown as the means ± SD (*n*=3). **(B)** Cell viability of indicated cells with or without cisplatin (CP) treatment for 48 h. Data are shown as the means ± SD (*n*=3). **(C)** Analysis of DNA damage and repair rate in indicated cells was conducted through the immunofluorescence staining of p-H2AX during recovery period following 12 h of Dox 1 µM or 24 h of CP 10 µM treatment. p values were calculated using two-tailed unpaired Student *t*-test. *p < 0.05, **p < 0.01, and ***p < 0.001.

To determine whether the increased ISGF3 components in GLDC deficient cells mediate cellular response to DNA damage, we knock-downed expressions of STAT2 and/or IRF9 in GLDC depleted ACHN cells treated with Dox/or CP. And we observed that knockdown of STAT2/or IRF9 alone as well as double knockdown of STAT2 and IRF9 dampened Dox/or CP-induced cell death as evidenced by increased cell viabilities in GLDC deficient ACHN and Caki-2 cells (Fig. 6A-B and Fig. S2A). Knockdown of STAT2/or IRF9 alone as well as double knockdown of STAT2 and IRF9 in the GLDC depleted ACHN cells reversed earlier increase and longer lasting appearance of p-H2AX as observed in GLDC depleted cells treated with Dox/or CP (Fig. 6C and Fig. S2B). These results demonstrate that ISGF3 mediates enhanced DNA damage and slower DNA repair in GLDC deficient cells.

### Knockdown of GLDC attenuates p53-dependent cell cycle checkpoint response to DNA damage and increases mitotic catastrophe

**Figure 7:**
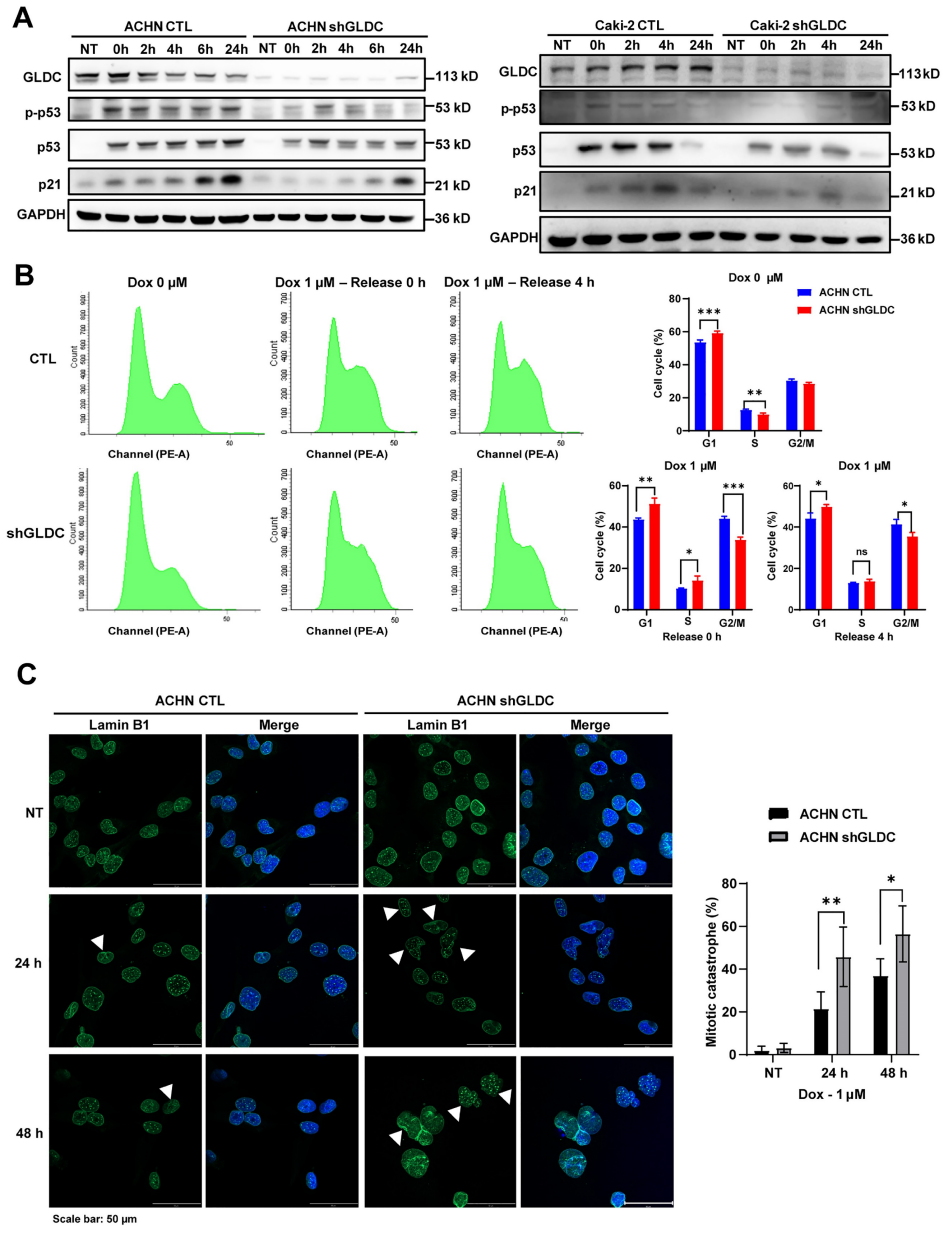
** Knockdown of GLDC attenuates p53-dependent cell cycle checkpoint response to DNA damage and increases mitotic catastrophe. (A)** Western blot of indicated proteins in ACHN and Caki-2 control (CTL) and GLDC knock-downed GLDC (shGLDC) cells during recovery period following the treatment of doxorubicin (Dox) 1 µM for 12 h. NT, non-treated cells.** (B)** Cell cycle analysis was performed by FACS in indicated cells treated with Dox for 12 h. Samples were collected during the recovery period, either 0 h or 4 h after treatment. Data are shown as the means ± SD (*n*=3).** (C)** Mitotic catastrophe was visualized by multi-nucleation through the immunofluorescence staining of Lamin B1 after treatment of Dox for 24 h or 48 h. White triangles were used to indicate abnormal interphase cells. The number of cells undergoing mitotic catastrophe was quantified. Data are shown as the means ± SD (*n*=3). p values were calculated using two-tailed unpaired Student *t*-test. *p < 0.05, **p < 0.01, and ***p < 0.001.

DNA damage triggers cellular responses including cell cycle arrest and subsequent repair processes. DNA damage recruits sensor proteins for detecting DNA damage and activates p53 dependent pathway to arrest cell cycle to ensure further DNA repair 34. Here, we found in both ACHN control and GLDC knock-downed cells that phosphorylation of p53 and expression of p21 increased in response to Dox treatment (Fig. 7A). Interestingly, increased levels of phosphorylation of p53 and expression of p21, downstream target of p53, are markedly lower in GLDC knock-downed ACHN and Caki-2 cells than those in the corresponding control cells. Expression of p21 in control cells increased immediately after cells were released from 12 h Dox exposure and continued to increase until 24 h. In contrast, expressions of p21 in GLDC knock-downed ACHN and Caki-2 cells increased later than those in the corresponding control cells and the degree of increased expressions of p21 were lower in GLDC knock-downed ACHN and Caki-2 cells than those in the corresponding control cells. Subsequent cell cycle analysis revealed that cells arrested in G2/M phase are more frequently found in control cells than those in GLDC knock-downed cells after Dox treatment (Fig. 7B). Furthermore, we found that 24 h after Dox exposure, approximately 60% of control cells are found arrested in G2/M phase while little more than 20% of GLDC knock-downed cells are found arrested in G2/M phase (Fig. S3A). These results suggest that depletion of GLDC enables cells to evade cell cycle checkpoints so as not to secure enough time to repair damaged DNAs. Since damaged DNAs cause mitotic catastrophe and subsequently cell death, we then investigated mitotic catastrophe by performing lamin B1 IF staining to determine mononucleated (normal interphase) and micro-nucleated (abnormal interphase) cells. Indeed, we observed that the percentage of GLDC knock-downed cells in abnormal interphase increased following Dox-induced DNA damage (Fig. 7C and Fig. S3B), a result that corroborates cell cycle analysis.

### Suppression of GLDC inhibits RCC progression *in vivo*

**Figure 8:**
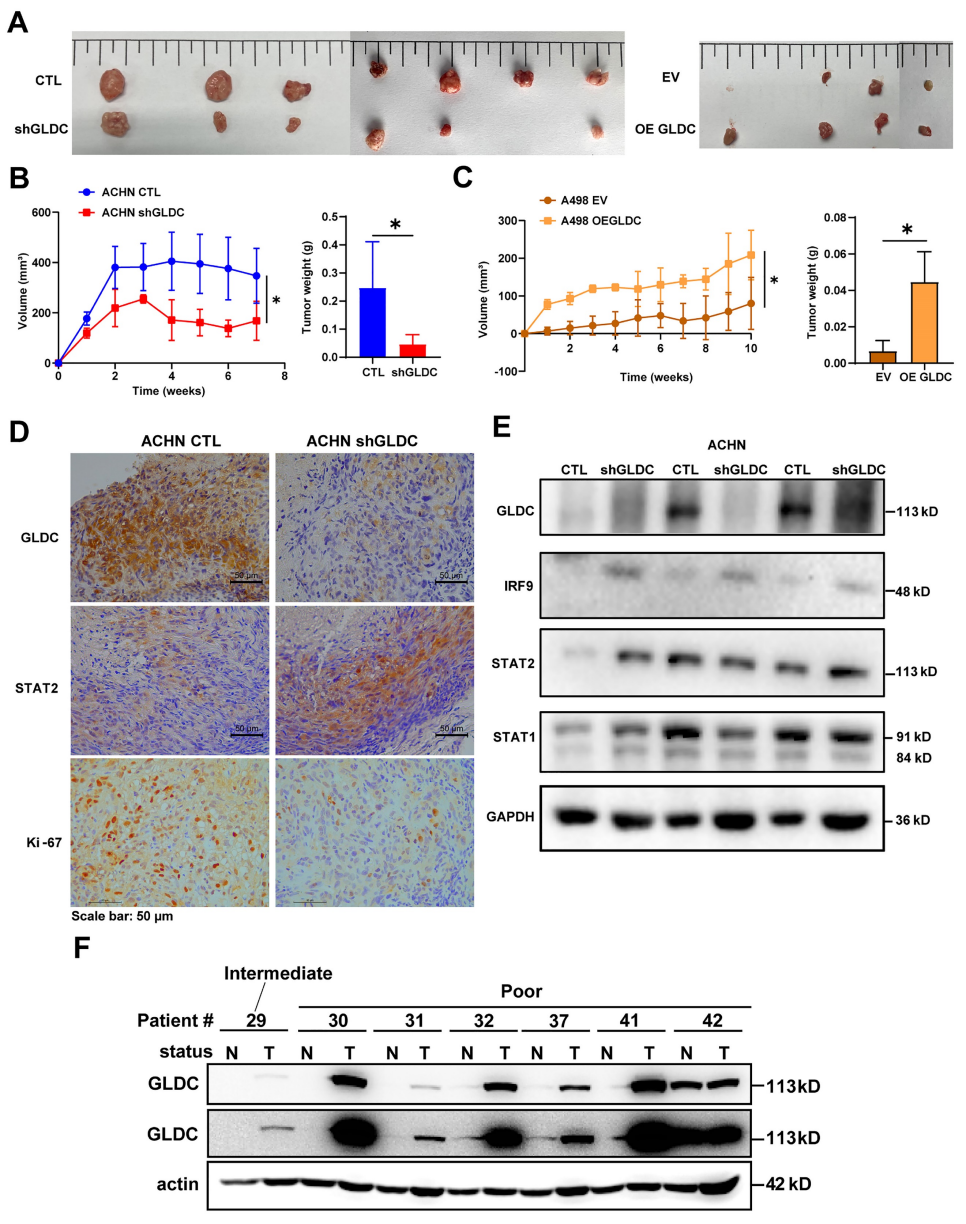
** Suppression of GLDC inhibits RCC progression *in vivo*. (A)** Images of xenograft tumor derived from injection of ACHN control (CTL) and GLDC knock-downed cells (shGLDC) (*n*=7) and A498 control (EV) and GLDC over-expressed cells (OE GLDC) (*n*=4). **(B)** Quantification of tumor volume and tumor weight from ACHN CTL and shGLDC cells. **(C)** Quantification of tumor volume and tumor weight from A498 EV and OE GLDC cells. **(D)** Immunohistochemistry staining of GLDC, STAT2, and Ki-67 in tumors injected with ACHN CTL and shGLDC cells. **(E)** Western blot of GLDC, IRF9, STAT2, STAT1, and GAPDH (loading control) in tumors injected with ACHN CTL and shGLDC cells. **(F)** Western blot of GLDC and actin (loading control) of tumor and non-tumor tissues from patients with high-risk RCC. p values were calculated using two-tailed unpaired Student *t*-test. *p < 0.05, **p < 0.01, and ***p < 0.001.

To prove and validate the effects of GLDC further, we xenografted GLDC knock-downed ACHN and GLDC overexpressed A498 cells subcutaneously into the flank of nude mice. We found that tumor volumes and weights of GLDC knock-downed ACHN cells were markedly lower than those of control ACHN cells while tumor volumes and weights of GLDC overexpressed A498 cells were markedly higher than those of control A498 cells (Fig. 8A-C). We then assessed the expressions of GLDC and ISGF3 in xenografted tumor tissues and confirmed decreased GLDC and increased IRF9 and STAT2 expressions (Fig. 8D-E). Ki67, a proliferation marker, also decreased in GLDC knock-downed ACHN tumors. Lastly, we explored the possibility of the *in vitro* and *in vivo* results of GLDC in the current study being translated into the treatment of human disease, especially RCC. We examined the expression levels of GLDC in the non-tumor and tumor kidney tissues from RCC patients with poor prognosis and found that expressions of GLDC were dramatically higher in the tumor tissues than those in non-tumor tissues, a finding that provides a novel therapeutic target for the treatment of RCC (Fig. 8F).

## Discussion

This study demonstrates that GLDC could be a potential therapeutic target for RCC. We identified that suppression of GLDC inhibits *de novo* nucleotide synthesis, which results in the accumulation of ROS. This accumulation of ROS in turn induces mitochondrial damage and stress, a cellular process that culminates in the attenuated proliferation of RCC cells and the decreased tumor growth. Conversely, overexpression of GLDC increased cell proliferation and tumor growth. Replenishment of dNTP in GLDC knock-downed cells lowered ROS accumulation and restored attenuated proliferation of RCC cells. Mechanically, downregulation of GLDC increases protein levels of IRF9 and STAT2, two components of ISGF3 subunit, and then activates the subset of ISGs. Also, silencing of IRF9 and STAT2 reverses decreased proliferation of RCC cells induced by downregulation of GLDC. In addition, we found that depletion of GLDC aggravates Dox and CP induced DNA damage via activating ISGF3-mediated pathway. Finally, we observed in the renal tissues of patients with poor prognosis that GLDC is highly expressed in tumors when compared with non-tumor tissues. These results collectively show that GLDC is oncogenic in RCC tumorigenesis and could be a promising therapeutic target for the treatment of RCC.

Little is known regarding the function of GLDC in tumorigenesis; moreover, the function of GLDC in tumorigenesis may seemingly vary in a cancer-type dependent manner. GLDC was first identified in non-small cell lung cancer as a factor critical to tumor initiation and overexpression of GLDC promotes cellular transformation and tumorigenesis 12, 35. The function of GLDC in the tumorigenesis of hepatocellular carcinoma (HCC) is conflicting. Previous studies suggested that GLDC may act as a tumor suppressor as evidenced by increased intrahepatic metastasis in GLDC knock-downed Huh7 orthotopic transplanted mice and attenuated intrahepatic metastasis in GLDC overexpressed HCCLM3 orthotopic transplanted mice 13, 36. A recent study, however, showed a slower growth of tumor in GLDC knock-out HepG2 grafted mice 37. These conflicting results regarding the function of GLDC in tumorigenesis in different HCC cells may implicate the function of GLDC in tumorigenesis may vary even in the same cancer-type. Herein, we show that GLDC acts as an oncogene in ACHN and Caki-2 RCC cells.

Previous studies identified that GLDC is a novel key gene in the progression and outcome of RCC. They showed that overexpression of GLDC suppressed proliferation, migration, and invasion of RCC 38-40. However, these results are not in line with those in our study. In contrast, we found that knockdown of GLDC, not overexpression of GLDC, inhibits cellular proliferation and tumor growth. One possible explanation for this conflicting result is that previous studies employed different RCC cell line, 786-O, with basal expression level of GLDC lower than other RCC cell lines, and our study used ACHN and Caki-2 with basal expression levels of GLDC higher than other RCC cell lines. These different effects of GLDC on tumorigenesis amongst RCC cell lines could be attributed to extensive tumoral heterogeneity and subclonal evolutionary nature of RCC 41.

Tumorigenic cells display hyperactive synthesis and use of nucleotides so as to support the uncontrolled proliferation and metastasis 10. Various oncogenes such as mutant KRAS, PI3K, and MYC involved in the nucleotide synthesis pathways have been discovered 42-44. Accordingly, inhibitors that selectively target the nucleotide synthesis pathways have been developed. However, except for 5-fluorouracil, drugs that target the nucleotide synthesis, especially pyrimidine synthesis, have largely been unsuccessful. One such notable example is Bre, a potent inhibitor of the enzyme DHODH. Despite its high potency and specificity, Bre failed all clinical trials due to severe adverse effects 45-47. GLDC is a part of glycine cleavage system (GCS) that donates one-carbon (1C) units to form 5, 10-methylene-THF, a molecule that is essential for nucleotide biosynthesis. GLDC is most highly expressed in the liver and then in the kidney 37. With this information, we hypothesized that GLDC could act as an oncogene in RCC via regulating nucleotide synthesis and regulation of GLDC might be a promising therapeutic strategy that could replace Bre. And indeed, we found that knockdown of GLDC decreased cellular proliferation and intracellular levels of dNTPs. Inhibition of nucleotide biosynthesis by Bre in RCC cells also decreased cellular proliferation, which was rescued by dNs treatment. Treatment of dNs into GLDC knock-downed cells restored cellular proliferation. These results suggest that GLDC may regulate cellular proliferation via modulating nucleotide synthesis.

Besides acting as a catalytic enzyme for glycine, GLDC is known to regulate ROS production 13, 36. Thus, we next exploited the possibility that GLDC-induced nucleotide depletion would lead to ROS production. And we found that GLDC knockdown and inhibition of nucleotide synthesis by Bre stimulated mtROS generation, a result that implicates decreased nucleotide synthesis induces ROS generation. Conversely, treatment of dNs significantly increased cellular proliferation and decreased mtROS generation in GLDC depleted cells. These results collectively suggest that inhibition of GLDC depletes dNTP cellular levels and the depleted dNTP levels in turn induce mtROS productions in RCC cells.

Excessive mtROS production is a major cause of DNA damage 48. Due to the proximity of the location to electron transport chain and absence of histones, mtDNA is more susceptible to ROS-induced damage than nuclear DNA. Excessive production of mtROS disrupts integrity of mtDNA, rendering mtDNA to be released into the cytoplasm and subsequently stimulating downstream pathways, such as stimulator of interferon genes (STING) and interferon (IFN)-related pathways. Intriguingly, a recent study identified GLDC as host susceptibility gene to severe influenza 31. They demonstrated that inhibition of GLDC markedly stimulated IFN-stimulated genes and also uncovered GLDC/pyrimidine biosynthesis/innate immune response axis. With this information, we reasoned that increased mtROS induced by GLDC inhibition causes mitochondrial stress and damaged mtDNA to be released into the cytoplasm, resulting in the activation of ISGF3 signaling pathway. And indeed, we observed activated ISGF3 pathway in GLDC knock-downed RCC cells as demonstrated by enlarged and aggregated nucleoids, a stereotypical phenotype of mtDNA-stress and increased expressions of STAT2 and IRF9, two components of ISGF3, and boosted expressions of ISGs. Further studies with double knockdown of IRF9 and STAT2 revealed that ISGF3 mediates the effect of GLDC on cell proliferation. The function of ISGF3 in the progression of cancer cells is controversial. While some studies suggest that ISGF3 stimulates tumor growth and exhibits chemotherapy-resistant effects 16, 17, others show that it suppresses tumor growth and enhances chemotherapy-induced damage 18, 28, 29. Herein, we found that increased ISGF3 induced by depletion of GLDC impairs proliferation of RCC cells, exacerbates CP-induced DNA damage, and decreases tumor growth in the mice.

## Conclusions

In this study, we demonstrated a regulatory role GLDC in the progression of RCC. We showed that GLDC deficiency diminished nucleotide biosynthesis, resulting in ROS generation. This increased production of ROS promotes mtDNA stress and activates ISGF3 pathway to inhibit progression of RCC cells and attenuate tumor growth in the mice. The results in the current study provide a scientific foundation based on which additional studies as to elucidate further the unveiled functions of GLDC. Further studies to develop a specific inhibitor of GLDC are also necessary. Given the finding that multiple tumor suppressors regulate a HIF-dependent negative feedback loop via ISGF3 18, GLDC could be a promising therapeutic target for the treatment of RCC.

## Supplementary Material

Supplementary figures and tables.

## Abbreviations

RCC: renal cell carcinoma
GLDC: glycine decarboxylase
IFN-I: interferon type I
SETD2: SET domain containing 2
H3K36me3: histone 3 lysine 36 trimethylation
ISGF3: interferon stimulated gene factor 3
U-ISGF3: unphosphorylated-ISGF3
STAT2: signal transducer and activator of transcription 2
IRF9: interferon regulatory factor 9
ISGs: interferon stimulated genes
ROS: reactive oxygen species
CP: cisplatin
dNTP: deoxynucleotide triphosphate
dNs: deoxynucleosides
dATP: deoxyadenosine triphosphate
dTTP: deoxythymidine triphosphate
dCTP: deoxycytidine triphosphate
dGTP: deoxyguanosine triphosphate
mtDNA: mitochondrial DNA
STAT1: signal transducer and activator of transcription 1
Bre: brequinar
DBSs: double-strand breaks
NT: non-treatment
Dox: doxorubicin
