# Supplementary material for: Glycine Decarboxylase Regulates Renal Carcinoma Progression via Interferon Stimulated Gene Factor 3-Mediated Pathway
Source: Int J Biol Sci. 2025 Jan 1;21(2):772–88. doi: 10.7150/ijbs.104458 (PMC11705630; doi:10.7150/ijbs.104458)
Supplement: Supplementary file 1 — Supplementary figures and tables. [file ijbsv21p0772s1.pdf]

Supplement materials and methods

Supplementary table 1. Primer sequences for real-time PCR

Primer name	Forward (5'-3')	Reverse (5'-3')
β-actin	GGACTTCGAGCAAGAGATGG	AGCACTGTGCCCGTACAG
GLDC	GCAGTTCACCTCAAGGCCAT	CTGAGCATTCATATTTGCCC
OAS1	GTTGCCACTCTCTCTCCTGT	CACCTTGGACACACACACAG
IFIT1	GGACAGGAAGCTGAAGGAGA	GCCCTTTTGTAGCCTCCTTG
IFI44	GAGGTCTGTTTTCCAAGGGC	CGCCTTCTTTCTCACTCAGC
IFI44L	GCCAAGTAAGCCCCATATGC	ATGGGATTTGAGGGCTTCCA
CCL5	GAAAGAACCGCCAAGTGTGT	GTGGTAGAATCTGGGCCCTT
MX1	TTGACGAAGCCTGATCTGGT	CTTCTCTCTCTGCAGGGCTT
IFI27	ACCAAGTTCATCCTGGGCTC	TTGGGATAGTTGGCTCCTCG

Supplementary table 2. DNA oligonucleotides used in dNTP assay

EvaGreen based detection	Sequences in 5' to 3' direction
Detection primer	CCGCCTCCACCGCC
dTTP detection template	TCGGTCCTCGCTCGCTCTTGCCTCGGTCCTTTATTT**GGCGGTGGAGGCGG**
dATP detection template	ACAGACCAGAGAGACACAACAGACGGAGGAAATAAA**GGCGGTGGAGGCGG**
dCTP detection template	TCAATCCCCACTCACTCTTACCTCAATCCTTTGTTT**GGCGGTGGAGGCGG**
dGTP detection template	TGAATGATGAGTGAGTGTGAGGTGAATGGTTTCTTT**GGCGGTGGAGGCGG**

Supplementary table 3. Short interfering RNAs (siRNA)

Gene name	Sequence
STAT2	GACUCUGGACAAUCUCAC<br>UGUGAGAUUGUCCAGAGUC
IRF9	CUCUUCAGAACCGCCUACU<br>AGUAGGCGGUUCUGAAGAG

Supplementary figures

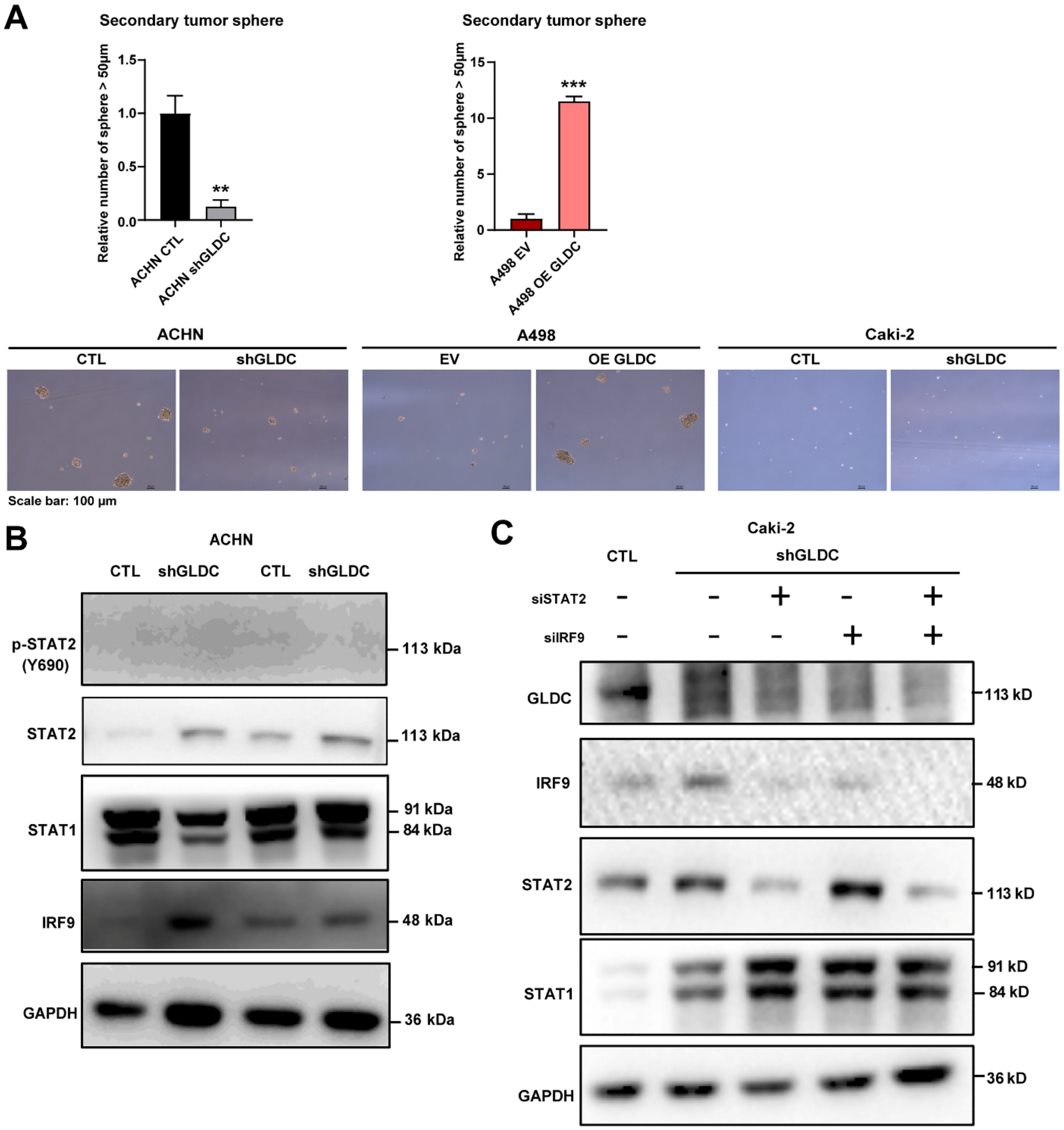

Figure S1.

(A) Secondary sphere formation of indicated cell lines. The number of spheres were calculated under microscope (*n*=3), and the sizes of spheres were determined using ImageJ.

(B) Western blot of p-STAT2, STAT2, STAT1, IRF9, and GAPDH (loading control) in ACHN control (CTL) and knock-downed GLDC (shGLDC) cells.

(C) Western blot of indicated proteins in Caki-2 CTL and shGLDC cells transfected with siRNA of negative control (NC), STAT2 and/ or IRF9.

p values were calculated using two-tailed unpaired Student *t*-test. *$p < 0.05$, **$p < 0.01$, and ***$p < 0.001$.

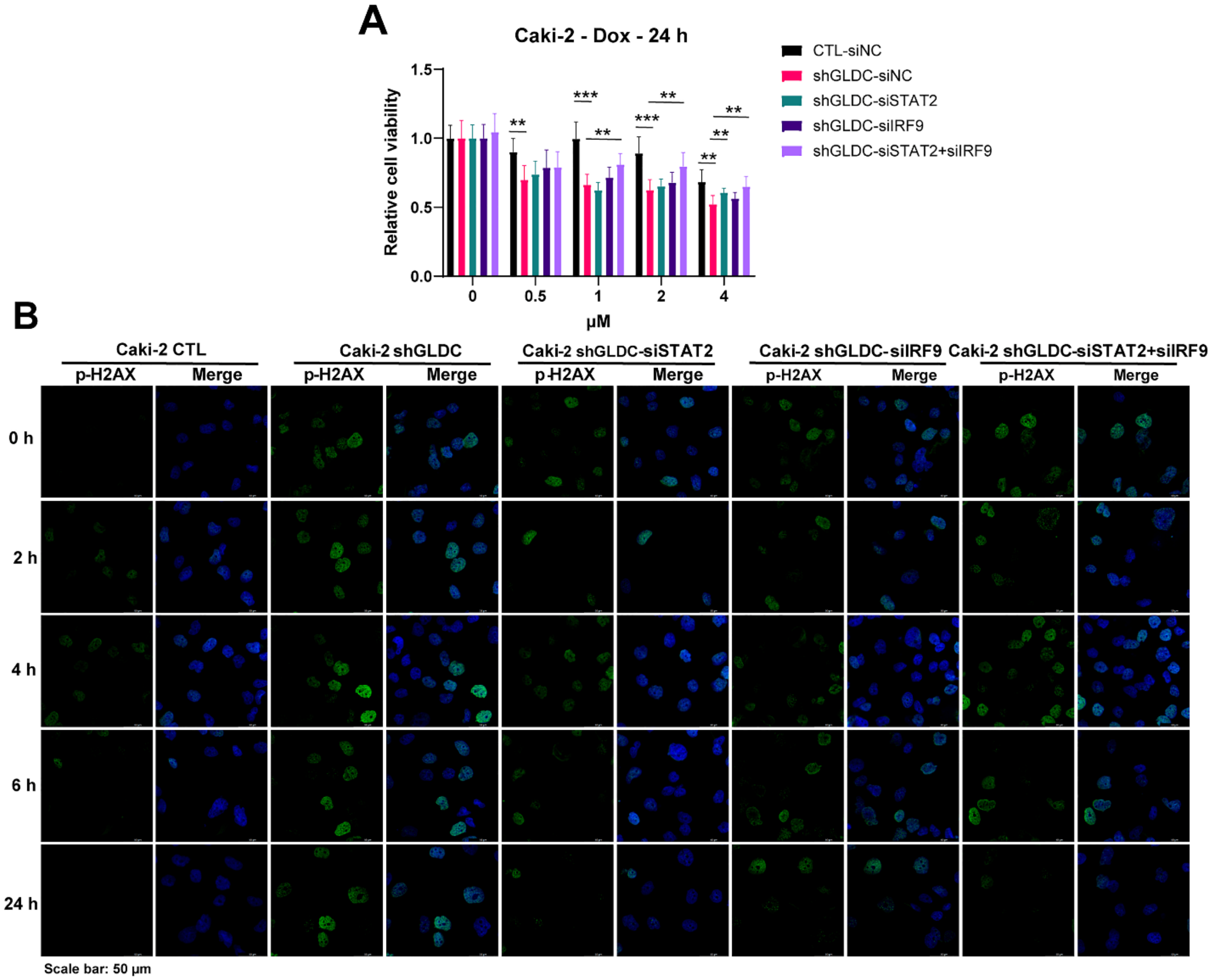

Figure S2.

(A) Cell viability of Caki-2 control (CTL) and knock-downed GLDC (shGLDC) cells transfected with siRNA of negative control (NC), STAT2 and/ or IRF9 and subsequently treated with doxorubicin (Dox) for 24 h (n=3).

(B) Analysis of DNA damage and repair rate in indicated cells was conducted through the immunofluorescence staining of p-H2AX during recovery period following 12 h of Dox 1μM.

p values were calculated using two-tailed unpaired Student t-test. *$p < 0.05$, **$p < 0.01$, and ***$p < 0.001$.

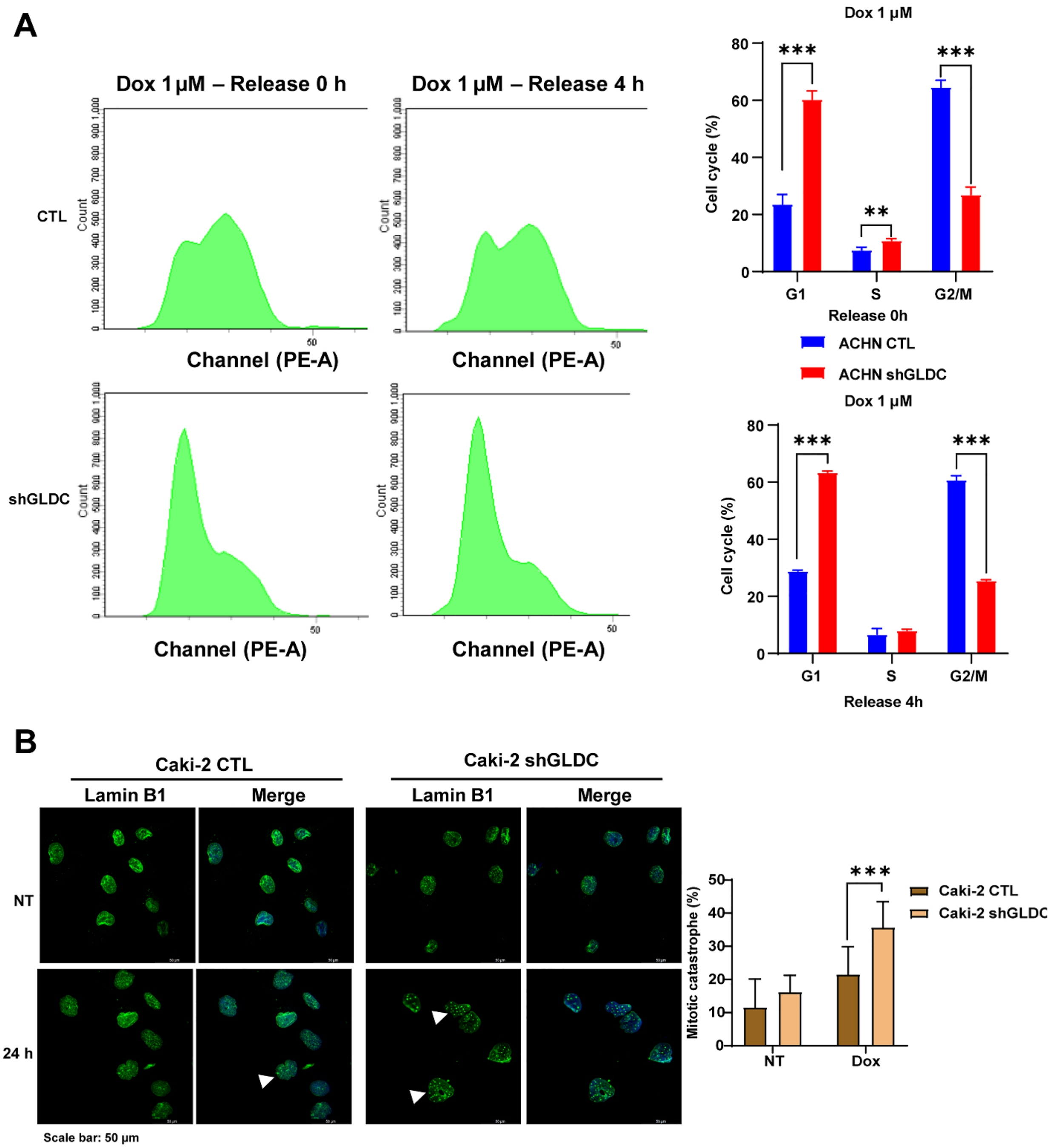

Figure S3.

(A) Cell cycle analysis of ACHN control (CTL) and knock-downed GLDC (shGLDC) cells after treatment with doxorubicin (Dox) 24 h, followed by the release of either 0 h or 4 h (n=3).

(B) Immunofluorescence staining of Lamin B1 in Caki-2 CTL and shGLDC cells after treatment of Dox for 24 h. White triangles were used to indicate abnormal interphase cells. NT, non-treated cells (n=3).

p values were calculated using two-tailed unpaired Student t-test. $*p < 0.05$, $**p < 0.01$, and $***p < 0.001$.
